# SVR12 rates higher than 99% after sofosbuvir/velpatasvir combination in HCV infected patients with F0-F1 fibrosis stage: A real world experience

**DOI:** 10.1371/journal.pone.0215783

**Published:** 2019-05-15

**Authors:** Alessandra Mangia, Valeria Piazzolla, Anna Giannelli, Egidio Visaggi, Nicola Minerva, Vincenzo Palmieri, Immacolata Carraturo, Domenico Potenza, Nicola Napoli, Gianfranco Lauletta, Vincenzo Tagarielli, Rosanna Santoro, Ernesto Piccigallo, Sergio De Gioia, Angelo Chimenti, Giuseppe Cuccorese, Antonio Metrangolo, Michele Mazzola, Ernesto Agostinacchio, Giuseppe Mennea, Carlo Sabbà, Marina Cela, Massimiliano Copetti, Ruggiero Losappio

**Affiliations:** 1 Liver Unit, IRCCS “Casa Sollievo della Sofferenza”, San Giovanni Rotondo, Italy; 2 Infectious Diseases Unit, Presidio Ospedaliero “Vittorio Emanuele II”, Bisceglie, Italy; 3 Internal Medicine Ospedale “Sarcone”, Terlizzi, Italy; 4 Internal Medicine “Ospedale Caduti in Guerra”, Canosa, Italy; 5 IInternal Medicine “A. Murri”, University of Bari, Bari, Italy; 6 Infectious Diseases Unit, Ospedale “V. Fazzi”, Lecce, Italy; 7 Interventional Ultrasound Unit, Ospedale “A. Perrino”, Brindisi, Italy; 8 Internal Medicine “C. Frugoni” University of Bari, Bari, Italy; 9 Internal Medicine “G. Baccelli” University of Bari, Bari, Italy; 10 Infectious Diseases Ospedale “F. Miulli”, Acquaviva delle Fonti, Italy; 11 Gastroenterology Division Ospedale “S. de Bellis” Castellana Grotte, Italy; 12 Internal Medicine Ospedale “SS Annunziata”, Taranto, Italy; 13 Infectious Diseases Ospedale “SS Annunziata”, Taranto, Italy; 14 Internal Medicine Ospedale “R.Dimiccoli”, Barletta, Italy; 15 Internal Medicine Ospedale “F. Ferrari”, Casarano, Italy; 16 Internal Medicine ASL BA, Bari, Italy; 17 Internal Medicine Ospedale “L. Bonomo”, Andria, Italy; 18 Gastroenterology “Ospedali Riuniti”, Foggia, Italy; 19 Geriatric Unit, Department of interdisciplinary Medicine, University of Bari, Bari, Italy; 20 Biostatistics Unit, Fondazione IRCCS Casa Sollievo della Sofferenza, San Giovanni Rotondo, Italy; Nihon University School of Medicine, JAPAN

## Abstract

**Background and objectives:**

The pangenotypic single tablet regimen of NS5B inhibitor sofobuvir (SOF) and NS5A inhibitor velpatasvir (VEL) is advised for 12 weeks in HCV-infected patients including those with compensated cirrhosis. Addition of ribavirin (RBV) may be considered in genotype 3 (GT3) with compensated and is recommended in decompensated cirrhosis. Real-life results with SOF/VEL are limited. To evaluate efficacy and safety in a large real-world-cohort including patients with different GTs and various fibrosis stages.

**Design:**

In total, 1429 patients were treated with SOF/VEL 400/100 mg for 12 weeks in the Puglia registry between June 2017 and May 2018. 1319 (92.3%) reached week 12 post-treatment (SVR12) at the moment. Only 41 received RBV. Diagnosis of cirrhosis was based on transient elastography and/or APRI or FIB-4 scores. Sensitivity analysis in the population including all patients except non virological failure was conducted. Primary efficacy endpoint was the percentage of patients with SVR12.

**Results:**

Patients’ mean age was 63.8 years, 42.3% had GT1. The majority were naïve and 735 (55.5%) F0/F2. Of the remaining 587, 282 had cirrhosis. SVR12 was 98.5%, 98.0% in GT1, 99.4% in GT2, 97.1% in GT3, 100% in GT4. Overall, SVR12 by sensitivity analysis was 99.4%; 99.7% among F0-F1. Among 218 PWID, SVR12 was 94.5%. Discontinuation rates were 3.7% among PWID and 0.7% among non-PWID (p = 0.004).

**Conclusions:**

SOF/VEL treatment of chronic HCV infection reaches very high cure rates in a variety of patients; including those with F0/F1 and PWID.

## Introduction

WHO guidelines aim at HCV elimination by 2030 [1]. The eradication objective is attainable through simple antiviral regimens, associated with high efficacy and universal duration, and therefore able to facilitate treatment access. In HCV treatment, real-world data validate the effectiveness and safety for regimens previously approved based on small numbers of patients. SOF/VEL is a Single Tablet Regimen (STR) (400/100 mg) administered for 12 weeks regardless of GT [2]. In phase III trials, this treatment demonstrates rates of SVR12 > 95% with excellent safety profile in patients with GT1-6 infection [3,4]. RBV addition is advised in GT3 cirrhotic and recommended in decompensated patients [5,6]. All other patients can be treated with a fixed 12-week regimen that does not require on treatment monitoring [6].

Current international guidelines [5,6] no longer recommend treatment prioritization, and patients with early stages of liver disease represent nowadays the largest group of treatment candidates—in particular among specific settings as people who inject drugs (PWID). PWID tend to be younger, with less advanced liver disease, and require rapid linkage to care and suitable treatment options in agreement with HCV elimination agenda. Real-life experiences with SOF/VEL regimen, in particular in patients with early stages of fibrosis are limited to preliminary reports including mainly GT2 and 3 patients [7,8].

It is still object of discussion, whether SVR12 rates are equally high in clinical trials and under real-world conditions regardless of fibrosis stages, genotype and population characteristics. Moreover, in real life, patients often bear co-morbidities and receive multiple medications leading to potential drug-to-drug interactions, making current HCV treatment more challenging than expected. SOF/VEL regimen was shown associated with no or limited interactions with other co-medications taken for co-morbidities [9].

In our multi center real world cohort, we aim to assess efficacy, safety and handling characteristics of 12 weeks SOF/VEL regimen ± RBV in patients infected with GT1-6 across all the fibrosis stages, considering possible drug-to-drug interactions. Adherence and treatment success rate in the subgroup of PWID were analyzed.

## Methods

For the present study, all consecutive patients with chronic HCV infection who completed SOF/VEL treatment between June 2017 and May 2018 at the participating centres in Puglia were included. The study group involves 19 of 31 regional prescribing centres sharing an ongoing program on DAA treatment since 2015. Of 1429 patients treated, 1319 who have reached week 12 post-treatment are included in this real-world-cohort analysis. The individual patient treatment schedule was chosen at the discretion of treating physicians [6]. In case of cirrhosis, GT3 infection or past treatment failure, RBV was administered when judged necessary. Patients who had failed SOF/RBV or SOF/NS3 inhibitor were included, patients with prior NS5A inhibitor therapy were excluded. With the exception of those with compensated cirrhosis and of PWID, patients were seen at baseline, week 4 on-treatment and week 12 post-treatment. Cirrhotic patients including decompensated and PWID received additional visits at week 8, at the end of treatment, and at 4 post-treatment. Demographic, clinical and virology parameters were assessed at each visit. Data were collected in an electronic database. Diagnosis of fibrosis stage was based on liver biopsy or on transient elastography (FibroScan, Echosens, Paris, France) (TE) results using the standard threshold of 12.5 KPa to define cirrhosis and 10.1 to define advanced fibrosis. Aspartate/platelets ratio index, APRI score and FIB-4 were available for every patient. A cut-off of >2, and >3.25 respectively were used to define cirrhosis [10].

## Study endpoints

The primary endpoint of this study was an undetectable HCV-RNA 12 weeks post-treatment (SVR12) assessed in he population including all patients except non-virological failure. Additional endpoints were adverse events rates (AEs) and treatment discontinuations due to AEs. SVR12, treatment emergent AEs and laboratory abnormalities were analyzed by fibrosis stage. Virological failure or relapse was defined as end of therapy undetectable HCV-RNA which became detectable again without proven re-infection.

The safety analysis included all patients who started SOF/VEL treatment, in whom at least a baseline visit was documented (overall cohort). Severe AEs including death or pre-terminal cessation of the medication due to AEs were assessed.

## Statistical analysis

SVR12 were analyzed in the whole population: all patients followed-up 12 weeks minimum, including patients who had treatment discontinuation for any reason or death during treatment or within 12 weeks of follow-up. A sensitivity analysis was conducted in the population including all patients except non virological failure. Patients who have not reached scheduled week 12 follow-up were not eligible for both analyses.

We performed further subgroup analyses on outcome within patients with F0-F1, F0-F2, F3-F4 and cirrhosis. Patients taking or not concomitant medications were also analyzed as subgroups, as well as patients with past or current substance use. For statistical analysis, Pearsons’s Chi Square test and Fisher’s exact test for categorical variables and t-test or ANOVA for continuous variables were used. A p- value <0.05 was considered statistically significant. All statistical analyses were performed using SPSS version 22.0 (IBM Corporation, New York, NY, USA).

## Results

### Baseline characteristics

A total of 1319 HCV infected patients from the entire population of patients treated in Puglia consecutively began SOF/VEL therapy for 12 weeks with or without RBV between June 2017 and May 2018 and were included in this cohort.

Detailed baseline characteristics of overall cohort and subgroups of patients ± significant fibrosis including liver function test are reported in Table 1. Median age of overall cohort was 63.8 years (range 18–92), 58.8% were male. Overall, 218 (16.5%) subjects were PWID. Most patients were naïve (80.2%). A total of 587 patients (44.5%, 95% CI 41.8–47.2) had advanced fibrosis (F3) or cirrhosis (F4). Cirrhosis was diagnosed in 282 (21.4%). Most patients had Child-Pugh-Turcotte (CTP) score A. Of 282 cirrhotic patients 22 had HCC (7.8%), thirteen (4.6%), had ascites during treatment.

**Table 1:** Baseline characteristics of patients.

Variables	Overall N = 1319	F0-F2 N = 732	F3-F4 N = 587	PWID N = 218
**Median Age, yrs (range)**	63.8 (18–91)	62.6 (18–91)	65.2 (20–91)	48.4 (18–66)
**N pts older than 65 yrs (%)**	668 (50.6)	353 (49.0)^	315 (53.6)	2 (0.9)
**N pts between, 18 and 65 yrs (%)**	651 (49.4)	379 (51.7)	272 (46.3)	216 (99.0)
**Males N, (%)**	776 (58.8)	405 (52.2) ^	371 (63.2)	187 (85.8)
**Females N,(%)**	543 (41.2)	327 (44.7)	216 (36.8)	31 (14.2)
**BMI ≥30 N (%)**	256 (21.3)	136 (20.0)	120 (23.3)	21 (9.6)
**BMI <30 N (%)**	1063 (80.5)	596 (81.4)	467 (79.5)	197 (90.3)
**Naïve N (%)**	1058 (80.2)	593 (81.0)	465 (79.2)	161 (73.9)
**Peg-IFN/RBV or SOF failures N (%)**	261 (19.8)	139 (19.0)	122 (20.8)	57 (26.1)
**Mean Liver Stiffness measure,** **KPa ± SE**	10.21+/-0.33	6.40 +/-0.95	15.39+/-10.54	12.4+/-1.07
**PWID N (%)**	218 (16.5)	116 (15.8)	102 (17.4)	NA
**RBV yes N (%)**	41 (3.1)	2 (0.3)	39 (6.6)	19 (8.7)
**RBV no N (%)**	1278 (96.9)	730 (99.7)	548 (93.4)	199 (91.3)
**Fibrosis stage N (%)**				
**F0-1**	335 (25.4)			23 (10.6)
**F2**	397 (30.1)			93 (42.7)
**F3**	305 (23.1)	NA	NA	55 (25.2)
**F4**	282 (21.4)			47 (21.6)
**HCV genotype N (%)**				
**1**	558 (42.3)	313 (42.8)	245 (41.7)	101 (46.3)
**2**	512 (38.8)	304 (41.5) ^	208 (35.4)	16 (7.3)
**3**	204 (15.5)	87 (11.9) ^	117 (19.9)	88 (40.4)
**4**	44 (3.3)	27 (3.7)	17 (2.9)	13 (6.0)
**5**	0	0	0	0
**6**	1 (0.1)	1 (0.1)	0	0
**Mean HCV RNA levels IU/ml (range)**	3.426.800 (15–7.810.000)	3.559.700 (16–7.810.000)	3.249.200 (33–4.700.000)	2.835.300 (21–4.324.000)
**Mean Creatinine mg/dl, range**	0.80 (0.2–2.10)	0.81 (0.9–2.10)	0.83 (0.6–1.95)	0.78 (0.7–1.5)
**Mean PLT count, 103/mm**^**3**^	210.1+/-21.6	212.7+/-48.6	199.2+/-23.0	186.6+/-67.2
**Mean Albumin g/dl ± SE**	3.87 +/-0.26	3.95+/-0.03	3.78 +/-0.04	3.98+/-0.81
**Child-Pugh-Turcotte A5 N, (%)**	231 (17.5)	NA	231 (81.9)	34 (15.6)
**A6 N, (%)**	32 (2.5)		32 (11.3)	6 (2.8)
**B N. (%)**	19 (1.4)		19 (6.7)	7 (3.2)
**Mean APRI score ± SE**	1.19 +/-0.07	0.74 +/-0.04	1.77 +/-0.15	1.21 +/-1.33
**Mean FIB-4 score ± SE**	3.05+/-0.16	2.11 +/-0.10	4.27 +/-0.32	2.39+/-2.71

^p<0.005

When characteristics of patients with different liver disease severity were analyzed, patients aged 18–66 were numerically more frequent among F0-F2 than among F3-F4. (51% *vs* 46%, p = 0.08). and Female were more frequent among F0-F2 than among F3-F4 (44.3% *vs* 40.0%, p = 0.01).

**Fig 1:**
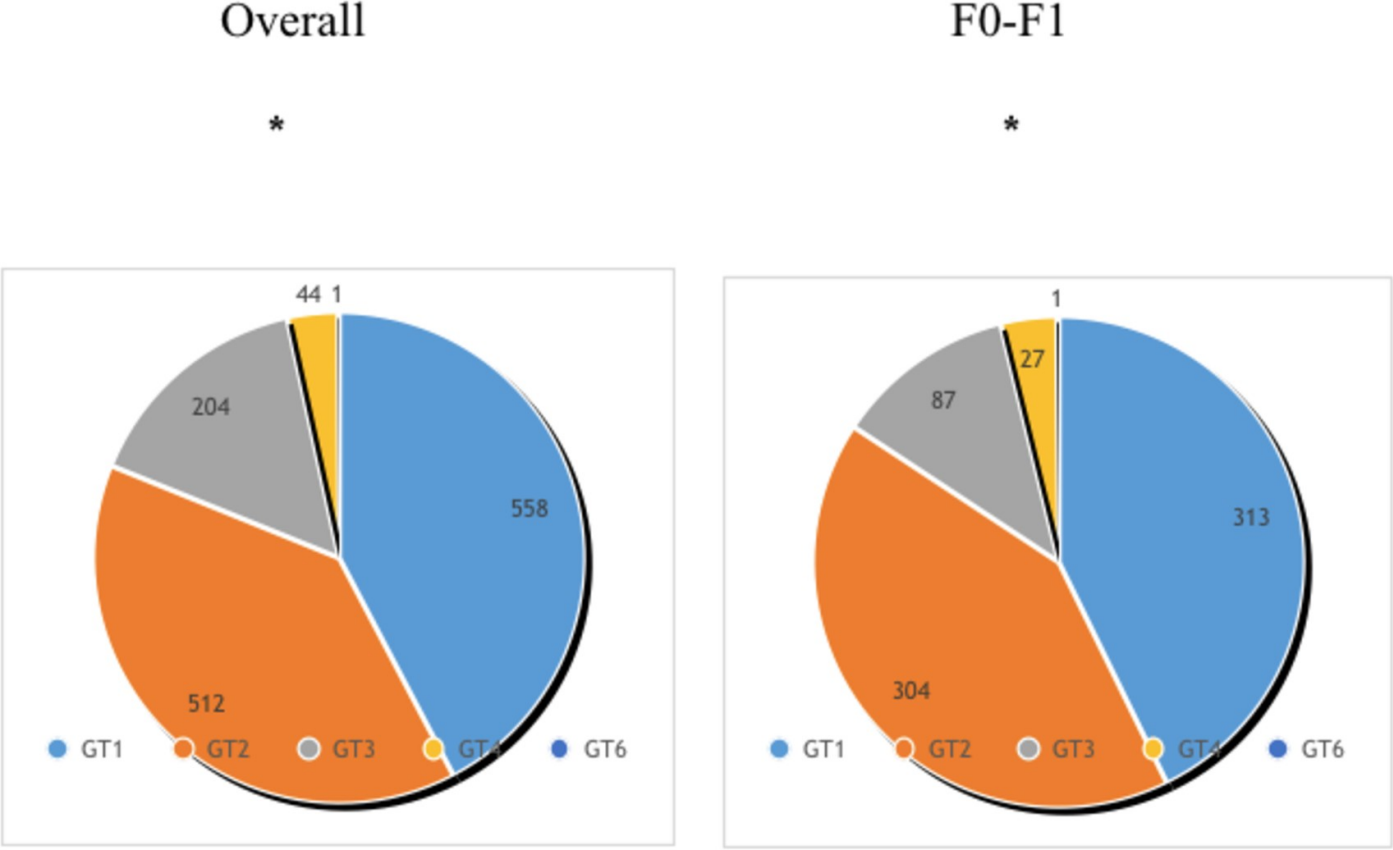
HCV genotype distribution overall and by F0/F1. The figure demonstrates that all the genotypes but GT5 are represented in this real world cohort. GT1 and GT2 were the most frequent accounting for 42% and 39% respectively. This proportion was maintained when 732 patients with F0-F1 stage of fibrosis were analyzed separately (42.7% were GT1 and 41.5% GT2).

All HCV genotypes but GT5 were represented (Fig 1). At baseline, genotype distribution was different between patients with F3-F4 and F0-F2 (p = 0.001), mostly due to uneven distribution of GT3. Of 204 GT3, 57.4% had stage F3-F4 as compared to 42.6% with F0-F2 (p = 0.70).

Treatment naive were 79% among F0-F2 and 81% among F3-F4, respectively (p = 0.44). Of PWID, 53.2% were subjects with F0-F2 as compared to 46.8% with F3-F4 (p = 0.45). Diabetes and obesity were statistically less frequent among F0-F2 than among F3-F4 (43.7% *vs* 56.3%, p = 0.004 and 46.3% *vs* 53.7%, p = 0.015, respectively). Due to physician’s preferences, only 3.1% overall received RBV: 0.3% among F0-F2 and 6.6% among F3-F4 (p<0.0001).

## Efficacy outcome and safety analysis

**Fig 2:**
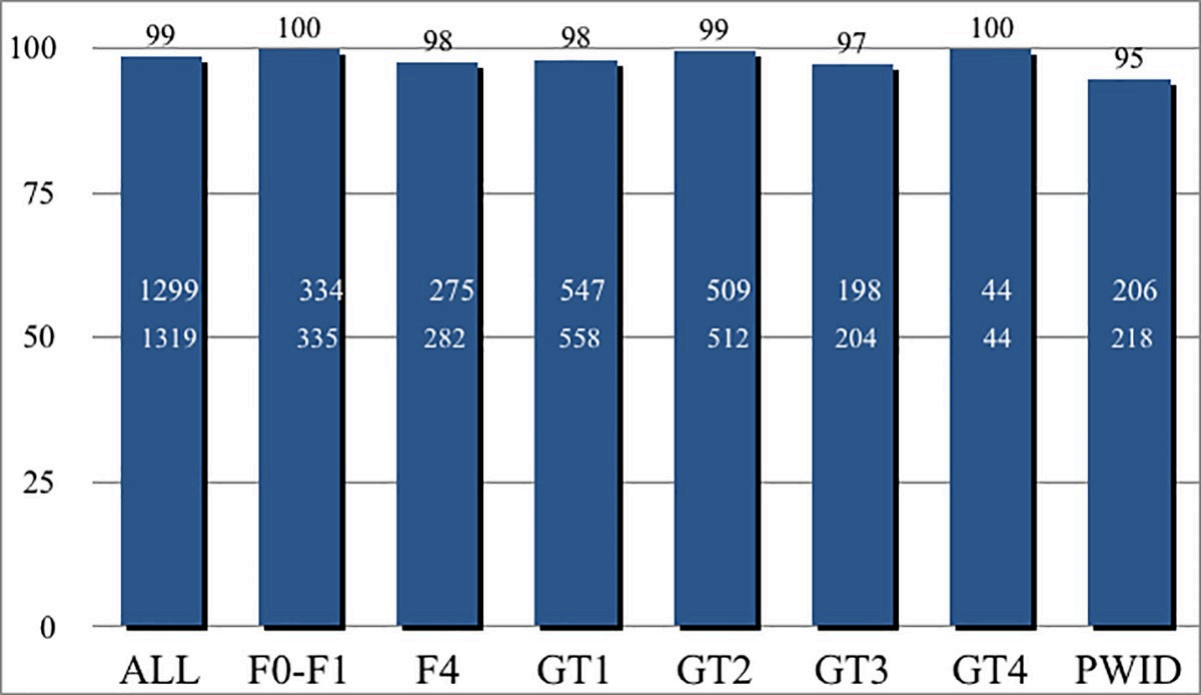
Proportion of patients with concomitant therapies. The figure shows the proportion of patients taking one ore more concomitant medications in this real world cohort. Only 191 patients (14.4%) were not taking concomitant medications. Up to 70% were taking 4 to 8 pill per day in addition to the antiviral treatment regimen.

Of the 1319 patients, only 20 did not achieve SVR12. In the whole population 98.5% (95% CI: 97.6%-99.0%) attained SVR12 after SOF/VEL. Of 20 patients who did not achieve SVR12, 8 were relapsers, the remaining had early discontinuation of treatment (n = 7) or were lost to follow up (n = 5). Study disposition in Table 2. Four additional patients who discontinued early achieved SVR12. Of 16 who were not able to complete treatment, two patients discontinued after a single dose. Among the 8 relapsers, 2 were proven re-infected, therefore the real SVR12 rate in the overall cohort was 98.7%, 95% CI: 97.8%-99.1%). The addition of RBV did not improve SVR12 that was 92.7% (95% CI: 78.9%-98.0%) with, and 98.7% (95% CI: 97.8%-99.2%) without RBV (p = 0.02). SVR12 was 98.3% (95% CI: 96.7%-99.1%) in 587 patients with F3-F4 and 98.6% (95% CI: 97.4%-99.3%) in patients with stage F0-F2 (p = 0.65). Analysis by genotype showed SVR12 of 98.0% in GT1, 99.4% in GT2, 97.1% in GT3, 100% in GT4 (p = 0.12). SVR12 by baseline characteristics is reported in Fig 2. Of 204 patients with genotype 3 only 25 received ribavirin in addition to SOF/VEL. SVR12 was 96% (95% CI 77.6%-99.7%) in patients treated with and 97.2% (95% CI 93.6%-98.8%) in patients treated without ribavirin (p = 0.54).

**Table 2:** Concomitant medications.

	Overall N = 1319	Male N = 776	Female N = 543
No concomitant medications	191 (14.4)	110 (14.1)	81 (14.9)
PPI N, (%)	699 (52.9)	466 (60.1)	233 (42.9)
NSAIDs N,(%)	661 (50.1)	309 (39.8)	352 (64.8)
Analgesics/opioids N, (%)	264 (20.0)	155 (19.9)	109 (20.0)
Lipid lowering agents N, (%)	254 (19.2)	147 (19.2)	107 (19.7)
Anti-diabetics N, (%)	244 (18.4)	158 (20.3)	86 (15.8)
Antihypertensive drugs N, (%)	207 (15.6)	114 (14.6)	93 (17.1)
Oral contraceptives N,(%)	97 (7.5)	NA	97 (17.8)
Antineoplastic N, (%)	43 (3.2)	22 (2.8)	21 (3.8)
HART/HBV N, (%)	11(0.8)	6 (0.7)	5 (0.9)
Iron chelators N,(%)	7 (0.5)	3(0.4)	4 (0.7)

## SVR by fibrosis stage

Among patients with fibrosis stage F3-F4, in the whole population SVR12 was 98.3%. The rate is similar to that observed in 732 patients with stage F0-F2 (p = 0.65). Of 20 patients who did not achieve SVR12, 10 had F3-F4 and 10 had F0-F2. More detailed analysis of SVR12 by stage of fibrosis demonstrated that 334 of 335 F0-F1 patients achieved SVR12 (99.7%). The corresponding figures in patients with F2 stage of fibrosis were 388 of 397 (97.7%, 95% CI: 98.0%-99.9%) (p = 0.026) (Fig 2). Virological responders for F3 and F4 were 99.0% (95% CI: 96.9%-99.7%) and 97.5% (95% CI: 94.7%-98.9%), respectively (p = 0.20). Excluding two re-infections, in stage 3 and 1 respectively, only 18 subjects experienced a relapse.

Mean APRI score for patients with SVR12 was 1.14. It was significantly higher than the corresponding 3.32 result of virological failure patients (p = 0.001). Using the APRI threshold of 2, 97.6% (95% CI: 96.0%-98.6%) of patients with result ≥2 achieved SVR12 which was not different from the 97.7% (95% CI: 91.9%-99.6%) attained by patients with lower APRI results (p = 1.0).

Analysis by fibrosis was performed also using FIB-4 score with a threshold of 3.25 to define cirrhosis. Patients with FIB-4 ≥ 3.25 achieved SVR12 in 97.5 (95% CI: 94.3%-99.0%) of cases. Patients with lower FIB-4 results achieved SVR12 in 97.7% (95% CI: 95.9%-98.7%) of cases (p = 1.0).

## Sensitivity analysis

Sensitivity analysis was performed on the entire cohort excluding patients who did not complete the assigned treatment for reasons not related to treatment. Overall, SVR12 was 99.1% (95% CI: 98.7%-99.7%). When patients with F0-F2 stage of fibrosis were analyzed, of the 10 non responders, 6 had discontinued treatment with a resulting SVR12 of 99.4% (95% CI: 98.4%-99.8%).

When patients were analyzed stage by stage, SVR12 in F0-F1 increased to 100%. The single patient who failed to achieve SVR12 had not completed week 12 post-treatment monitoring, but had achieved SVR4. By sensitivity analysis SVR12 in F3 and F4 was identical, 99.3% with 95% CI: 97.3%-99.8% for F3, and 97.1%-99.8% for F4, respectively.

Analysis of data by FIB-4 and APRI confirmed SVR12 by TE.

## SVR12 by past treatment experience

History of prior treatment might influence SVR12 rates. In this study, only 261 patients were treatment-experienced; of them 231 had failed Peg-IFN-based, 30 SOF-based regimens (including the combination with simeprevir or ribavirin). In the whole population, SVR12 rate among treatment failures was 98.5% (95% CI: 95.8%-99.5%), which was identical to that observed among naïve patients (p = 1.0); this suggests that with SOF/VEL a previous negative treatment result, including protease inhibitors failure is not anymore a negative predictor. When a subgroup analysis by stage of fibrosis and past treatment history was performed, within naive SVR12 rates (98.5%) were identical for F0-F2 and F3-F4. For experienced patients, 97.5% (95% CI: 92.4%-99.36%) SVR12 rate associated with F3/F4 stage was marginally lower than 98.5% (95% CI: 95.4%-99.9%) of patients with F0-F2 (p = 0.34).

Finally, SVR12 were analyzed by prior treatment and genotype. SVR12 were not statistically different across the different genotypes. Only among GT3, SVR12 was numerically lower for previously treated as compared to naïve patients (98.2%, 95% CI: 94.4%-99.5%*vs* 91.2%, 95%CI: 76.4%-97.8%, p = 0.069).

## Baseline characteristics and SVR12 among PWID

Overall, 218 (16.5%, 95% CI 14.6–18.6) PWID patients were treated within this real-world-cohort. Of them, one with HCC moved to another Country and discontinued after the first dose. Overall, 40% were on OST, 60% on active drug use having injected drugs during the previous month (mostly heroin). Patients on OST were taking methadone (n = 69) or buprenorphine (n = 18). The vast majority of PWID (85.8%) were male. Male gender was significantly more common than in nonPWID (p = 0.0001). Over 95% of patients were younger than 66 years, this proportion was also significantly higher than in nonPWID (p<0.0001). Only 4 were HIV antibodies positive with undetectable virus on stable treatment for HIV. Within PWID 53.2% had F0-F2 stage of fibrosis as compared to 55.9% in nonPWID (p = 0.45). Of patients with CTP B7-9, 7 were PWID. The proportion of patients with GT3 was significantly higher than in general population (43%*vs*10.6%, p = 0.0001). Study disposition in PWID and nonPWID is reported in Table 3. Overall, 94.5% (95% CI: 90.3%-97.0%) of PWID achieved SVR12 as compared to 99.3% (95% CI: 98.5%-99.6%) among not PWID (p<0.0001). By sensitivity analysis, SVR12 rates rose to 97.6% (95% CI: 94.2%-99.9%) as of 12 patients who failed to achieve SVR12 in this subgroup, 4 were lost-to-follow-up and did not achieve end of treatment, 5 experienced a relapse,and 3 died.

**Table 3:** Study disposition in general population and PWID.

Patients, n (%)	Overall n = 1319	Non PWID n = 1101	PWID n = 218
Completed drug N (%)	1300 (98.5)	1090 (99.0)	210 (96.3)
Discontinued N (%)	16 (1.2)	8 (<1)*	8** (3.6)
AE N (%)	0	0	0
Lost to follow-up N (%)	4 (0.3)	0	4 (2.0)
Noncompliance N (%)	0	0	0
Withdrew consent N (%)	0	0	0
Death N (%)	8 (0.6)	5 <1)	3(1.3)
Lack of Efficacy N (%)	8 (0.6)	3 (<1)	5 (2.3)

* A single patient who discontinued prematurely at week 8 achieved SVR12

**3 patients achieved SVR12 despite a premature discontinuation

Both reinfections by different genotype were observed among PWID. Although by sensitivity analysis 97.6% SVR12 remained significantly lower than 99.7% (95 CI: 99.1%-99.9%) rate observed in nonPWID (p = 0.004), discontinuation rates among PWID resulted comparable to those previously reported in ad-hoc studies [11].

## SVR, co-morbidities and concomitant medication

**Fig 3:**
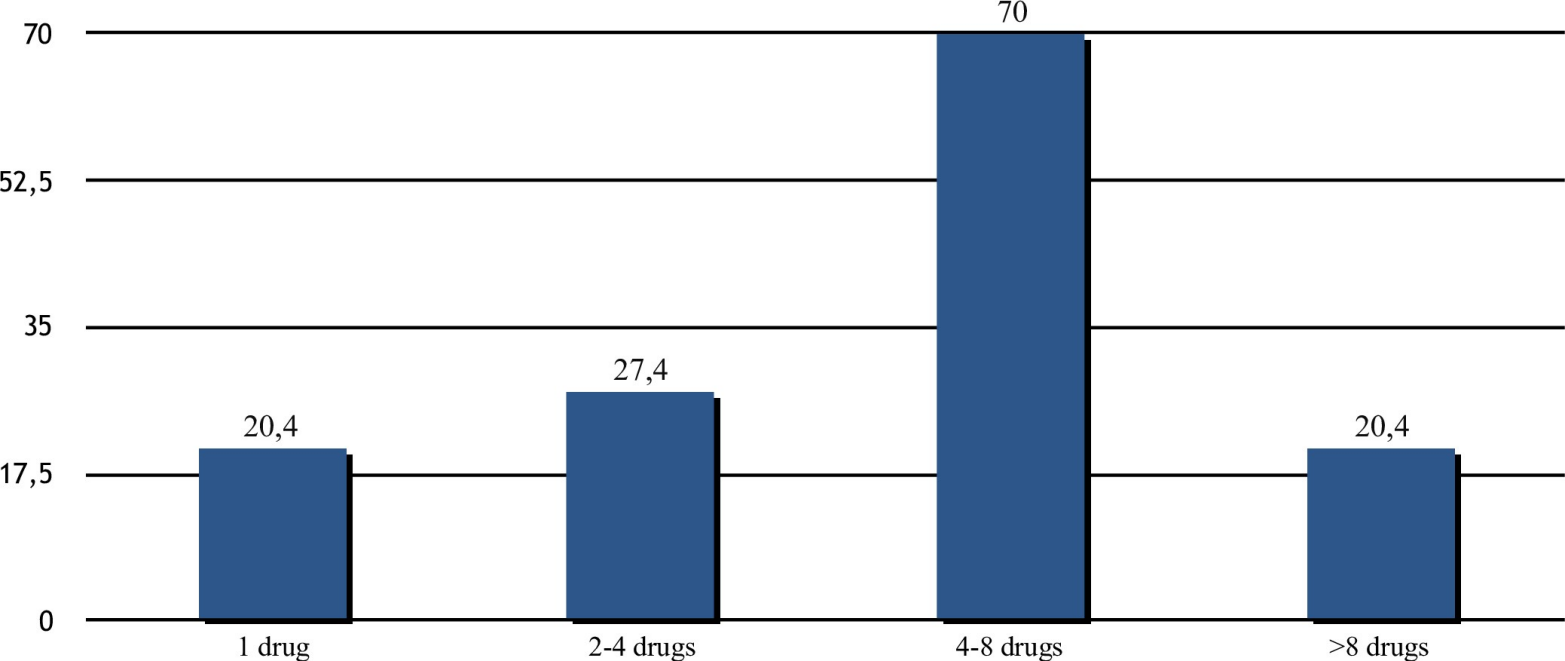
SVR12 overall and by baseline characteristics. SVR12 were extremely high regardless of baseline characteristics of patients including fibrosis stages, HCV genotypes and key populations including PWID.

A wide range of concomitant diseases was present in this Real-Word HCV cohort and as shown in Table 2 only 14.4% of patients were not taking concomitant medications. As reported in Fig 3, 70% of patients taking concomitant medications received from 4 to 8 different drugs in association with SOF/VEL. All patients with a neoplastic disease received concomitant chemotherapy without any additional adverse event and were able to complete their oncologic schedule. Of 228 (15.3%) patients with blood hypertension 91% were receiving various antihypertensive medications including sartans, enalapril and amplodipine. Ivabradine and NAO (dabitagram, apixabam and clopidogrel) were well tolerated. Beta-blockers were used by 21 patients no side effects were reported. No patients on amiodarone were included. Two hundred and fifty four patients were taking lipid lowering agents; only those taking atorvastatin and rosuvastatin were discontinued for 12 weeks to be re-started after antiviral therapy. Treatment with simvastatin was continued at 20 mg. Moreover, 97 of 303 female patients in their active sexual life were taking oral contraceptives and carried on without any negative effect on SVR12 rates. Of 20 HBV infected patients, 11 were already on treatment with entecavir (n = 6) and tenofovir (n = 1) and had undetectable HBV-DNA levels. The patient on tenofovir showed no relevant kidney or bone metabolism change during follow-up. The remaining 7 patients had inactive HBV infection and mild liver disease. None of the 4 patients on concomitant HIV treatment, including protease, nucleotide reverse transcriptase and integrase inhibitors required any HAART modification. Among PWID, 35% of patients were taking different analgesic (10 olanzepine, 15 haloperidol and 21 ketamine). None of them experienced related toxicity. Additionally, 14 patients on alprazolam, 10 on diazepam, 4 on zolpidem, continued their treatments. Urological agents were used by 9% of male patients including tamsulosin, alfuzosine and dutasteride. No toxicity was observed.

Diabetic patients were taking medications including sitagliptin, insulin and dulaglutide in 67% of cases, very few were on acarbose and the remaining on metformin. Their medications were not changed. All the patients with thalassemia major continued on their medications including oral deferasirox, deferiprone or hormone substitution therapy.

Twenty five patients had a chronic kidney disease, three discontinued treatment for reasons not related to treatment. All the remaining attained SVR12.

A substantial group (52.9%) was on proton pump inhibitors at baseline. Three hundred and forty three (49.3%) of them discontinued, the remaining continued their treatment according with the SOF/VEL package insert recommendations. Data on PPI use were not available for 167 patients [2].

## Efficacy in patients with decompensated cirrhosis/HCC

Our cirrhotic patients included 32 (11.3) on Child-Pugh-Turcotte A6 and 19 on Child-Pugh-Turcotte B7-9 (6.7%). RBV in addition of SOF/VEL was administered in no more than 11 of 19 Child-Pugh-Turcotte B patients. These patients were all male, 3 did not complete the assigned treatment course, 3 required RBV dose reductions. All but the three patients who discontinued RBV achieved SVR12. SVR12 was achieved by 353 of 358 (98.6%), (95% CI 96.7%-99.4%) patients in Child-Pugh-Turcotte A and by 16 patients in B (84.2%) (95% CI 62.4%- 94.4%) (p = 0.05).

Of 13 patients with on-treatment ascites, SVR12 rate was 76.9% (95% CI: 45.9%-93.8%) significantly lower than 98.6% (95% CI: 97.7%-99.1%) of patients without (p = 0.001). Among 21 patients with HCC, 17 (80.9%, 95% CI: 57.4%-93.7%) achieved SVR12. This rate was significantly lower than that 98.7% (95% CI: 97.9%-99.2%) of patients without HCC (p = 0.0001).

## Efficacy in patients using proton pump inhibitors (PPI)

Of 697 patients who were using PPI at the start of treatment, 354 (50.7%) continued their treatment according to SOF/VEL prescribing information. Three hundred and thirty eight (95.5%) of 354 patients who received PPI during treatment achieved SVR12. This rate was not different from the 97.8% rate attained in 622 patients who did not receive PPI (p = 0.48).

## Characteristics of patients who experienced a relapse

Characteristics of 20 patients who failed to achieve SVR12 are reported in Table 4. The majority of relapsers were male and naïve. Interestingly, 60% of patients who failed to achieve SVR12 was PWID. This finding highlights the need for closer and more frequent monitoring in PWID.

**Table 4:** Baseline characteristics of patients with virological failure.

Pt No.	Gender	Age	Geno type	Viral load	Stage	PWID	NS3/NS5 RASs	Tx history	Tx results
1	F	85	1b	162.409	4	No	No	Naïve	Discontinuation
2	F	43	1b	1.460.000	2	Yes	No	Naïve	Relapse
3	M	55	1a	134.500	4/HCC	Yes	No	Naïve	Discontinuation
4	M	51	3	10.756.000	2	Yes	No	Peg/IFN/RBV failure	Discontinuation
5	M	78	2	2.690.000	4	Yes	No	Naïve	Discontinuation
6	M	41	1b	3.541.000	2	Yes	Yes	Peg-IFN/RBV failure	Relapse
7	M	81	2	4658886	3	No	No	Naïve	Discontinuation
8	M	62	3	518943	4/HCC	Yes	No	Naïve	Discontinuation
9	F	46	3	791688	3	Yes	Yes	Naïve	Relapse
10	M	40	1a	580000	3	Yes	No	Naïve	Discontinuation
11	F	82	1b	122372	4	No	Yes	Peg/IFN/RBV Failure	Relapse
12	M	40	1a	352813	1	Yes	No	Naïve	Relapse
13	M	87	1b	431326	3	No	No	Naïve	Discontinuation
14	M	57	1b	1208	3	Yes	No	Peg/IFN/RBV Failure	Discontinuation
15	M	69	1a	12000000	2	No	No	Naive	Discontinuation
16	M	68	1b	3660000	4	No	No	Peg/IFN/RBV Failure	Relapse
17	F	79	2	1589300	2	No	No	Naive	Relapse
18	F	27	3	39700000	2	Yes	No	Peg/IFN/RBV Failure	Relapse
19	M	78	2	4205102	1	No	No	Peg/IFN/RBV Failure	Discontinuation
20	M	57	3a	385350	4	Yes	No	Naïve	Discontinuation

## Safety

Twelve patients prematurely discontinued treatment, and did not achieve SVR12, 7 of them died. Of the remaining, 4 were noncompliant PWID lost to follow up between week 8–12. One additional PWID with HCC—who died later—discontinued after one dose. Four additional patients only received 8 weeks of treatment but were able to achieve SVR. Two of them were PWID.

Of 8 deaths (0.6%), 4 were related to: acute myocardial infarction after treatment week-11, lung cancer in a F3 patient after treatment week-10, car accident at follow-up week-12 and HCC bleeding after day-1 of treatment.

In 4 more cases, death was due to worsening of decompensated liver cirrhosis and HCC in F4 fibrosis stage. Deaths were judged not treatment-related. AEs were reported in 75% of patients overall. Table 5 shows AEs by RBV use. Laboratory AEs were mostly RBV-related with Hb <10 mg/dl in 7 patients on RBV. Five of them required RBV dose reduction.

**Table 5:** AE in patients treated with SOF/VEL or with SOF/VEL/RBV.

	SOF/VEL (n = 1278)	SOF/VEL/RBV (n = 41)
Any AE, n (%)	969 (75.8)	30 (73.1)
*AEs leading to discontinuation*, *n (%)*	0	0
*Serious AEs*, *n (%)*	18 (1.4)	1 (2.4)
*Serious AEs possibly related to SOF/VEL*, *n (%)*	0	0
*AEs occurring in > = 5% of patients*		
Fatigue n(%)	907 (70.9)	36 (87.8)
Headache n(%)	881 (68.9)	34 (82.9)
Nausea n(%)	830 (64.9)	24 (58.5)
Anemia n(%)	5 (<1)	13 (31.7)
Diarrhea n(%)	40 (3.1)	11 (26.8)
*Laboratory*		
Hb<10 g/dl n(%)	4 (<1)	7 (17.0)
PLT <50.000 mm3 n(%)	17 (1.3)	7 (17.0)

## Discussion

This large real-world-cohort experience is the first on SOF/VEL enrolling comparable numbers of GT1, GT2 and GT3 patients. SOF/VEL without RBV results in 98.5% SVR12 rates in patients with different stages of fibrosis. SVR12 increases from 98.6% in patients with F0-F2 to 99.7% in F0-F1. By sensitivity analysis, SVR12 in patients with F0-F1 was 100%. Given that, the vast majority of patients currently seeking treatment have mild liver disease and SOF/VEL was used in our study without frequent monitoring, the results attained in F0-F1 patients support test-and-treat strategy with SOF/VEL. Among 282 cirrhotic patients, SVR12 was 97.5%. Our results mirror and improve on the results of registration trials [3,4]. Noticeably, this regimen did not require co-medication changes and cure rates were not affected by concurrent drugs including statins and PPI. Given the difference in potential for drug-to-drug interactions of different pangenotypic regimens [9]our results support the use of SOF/VEL whatever the co-morbidities or co-medications.

At variance with the first wave of HCV treatment, when patients were well engaged and motivated due to the long warehousing and possibly repeated prior failures, the patients currently treated are often new to HCV and less motivated to treatment [12–13]. Consequently, risk of low linkage to care, high discontinuation rates or lack of adherence is nowadays potentially increased. The advantage of a regimen that does not require changes in patients’ lifestyle and is associated with extremely high rates of treatment completion is remarkable. Our results demonstrate that working-adult candidates to HCV treatment can be treated and cured regardless of concomitant diseases and medications, and without any limitation in their daily life routine. The <1% discontinuation rate observed in our real-world experience (mostly due to lower adherence among PWID) is a major achievement outside of ad-hoc studies.

Real-world data using SOF/VEL from comparable large cohorts in the US have so far focused on GT2 and GT3 patients [7,8]. In another report including 900 patients treated with SOF/VEL±RBV only 155 were GT1 infected [14]. Among 567 patients, including those with cirrhosis enrolled in the TARGET study (28.5% of whom treated with RBV), overall SVR12 at PP analysis was 94.4% [15]. SVR12 rates were 96.6% for patients treated without RBV and 88.9% for those receiving RBV [15]. Data from the TRIO real-world-cohort were influenced by different access policies in the US, and therefore focused only on 84 patients with GT1 who achieved SVR12 in 97% of cases [14,16].

Glecaprevir and pibrentasvir (G/P) combination has been recently implemented with comparable efficacy. However, different treatment duration need to be adopted with this regimen on the basis of fibrosis severity and genotype [17]. Berg et other reported real-world data from the German registry on 638 naïve and IFN- or SOF/RBV-experienced patients, 96 of whom with SVR12 available. Patients had received G/P for 8 or 12 weeks [18]. Of 91 patients without cirrhosis treated for 8 weeks, 97% attained SVR12. AEs leading to treatment discontinuation were reported in 2% of patients, with 1% reporting either AST and total bilirubin increase [18]. Similar results were reported in 723 Italian patients who had SVR12 available after 8 or 12 weeks of G/P. In this study, a significantly lower response rate was registered among GT3 treated for 8 weeks [19]. Among 572 F0/F2 patients, SVR12 was 99.1%. Interestingly, a 3.5% rate of treatment discontinuations was registered after treatment duration of 12 weeks and deaths were reported in 2.4% of subjects.

At variance, SOF/VEL regimen in our study was not associated with laboratory AEs and drug-to-drug toxicity. Our results highlight how SOF/VEL one-size-fit-all strategy may dramatically increase access to high efficacy treatment, and display the excellent safety profile of this STR [19,20]. Consequently, even without estimating fibrosis, treatment with SOF/VEL can be started regardless of prior treatment history, once established that HCV is replicating.

About 20% of our patients are currently PWID [21], although the number of discontinuations/lost-to-follow-up rate was higher than in general population, the proportion of treatment completion was overall high. Either SVR12 (90.4%) or rate of treatment discontinuations were similar to those observed in clinical trials on patients on opioid-substitution treatment [11, 22–24]. Of note, at variance with studies using electronic blisters to ensure adherence of PWID patients, in our study, patients underwent a monthly visit associated with returning empty bottles and refilling. Similar SVR12 rates were shown more recently in HIV/HCV co-infected patients in Germany [25–27]. Strictly related to the PWID treatment is the issue of re-infection. In our study, only two re-infections were registered, representing less than 0.1% of general population and 1% of PWID. These results are in keeping with those of a combined analysis of phase III studies [28].

In patients with decompensated cirrhosis, real-world experience results are based on the TARGET cohort. In this cohort focusing on patients with MELD >10 treated with SOF/VEL, of about 100 patients who received SOF/VEL ± RBV, 90.5% achieved SVR12, 3% of patients died and one of two of decompensated patients progressed after SVR demonstrating that eradication of HCV does not mean increased survival in patients with very advanced liver disease or with severe co-morbidities [29]. In our study, SVR12 attained by patients in CTP B or higher was 84.2% and 3 deaths were reported in this subgroup. RBV addition was more likely to be in patients more prone to failure. How to identify among our patients seeking a cure who will continue to progress despite SVR12, remains not yet adequately clarified.

The current real-world experience is an observation cohort study with inherent limitations of cohort studies including the risk of selection bias. This risk was addressed by the inclusion of all the patients consecutively treated with SOF/VEL at each center participating in this experience.

In conclusion, our real-world-cohort study bears three key messages. SOF/VEL treatment for chronic HCV patients generates similar SVR12 rates—as those seen in the registration trials–which are higher than 95% regardless the use of RBV. SVR12 rates were not different across genotypes and fibrosis stages. Among PWID, SVR12 rates in real life resulted as high as in clinical studies, although we cannot exclude that a dedicated closer monitoring could have been associated with less lost-to-follow up or early discontinuations. This regimen provides prescribing physicians with the opportunity to increase treatment access and to achieve WHO HCV elimination 2030 objectives.
