# Correction: SVR12 rates higher than 99% after sofosbuvir/velpatasvir combination in HCV infected patients with F0-F1 fibrosis stage: A real world experience

**DOI:** 10.1371/journal.pone.0223287

**Published:** 2019-09-25

**Authors:** Alessandra Mangia, Valeria Piazzolla, Anna Giannelli, Egidio Visaggi, Nicola Minerva, Vincenzo Palmieri, Immacolata Carraturo, Domenico Potenza, Nicola Napoli, Gianfranco Lauletta, Vincenzo Tagarielli, Rosanna Santoro, Ernesto Piccigallo, Sergio De Gioia, Angelo Chimenti, Giuseppe Cuccorese, Antonio Metrangolo, Michele Mazzola, Ernesto Agostinacchio, Giuseppe Mennea, Carlo Sabbà, Marina Cela, Massimiliano Copetti, Ruggiero Losappio

In the SVR, co-morbidities and concomitant medication subsection of the Results, there is an error in the 15th sentence of the first paragraph. The correct sentence is: Among PWID, 35% of patients were taking different antipsychotics or anesthetics to treat chronic pain as analgesics (10 olanzepine, 15 haloperidol and 21 ketamine).
